# Pneumatosis coli secondary to lactulose use masquerading as an acute abdomen

**DOI:** 10.1093/jscr/rjaf165

**Published:** 2025-03-28

**Authors:** Mostafa Amer, John Woodfield

**Affiliations:** Department of Surgical Sciences, Otago Medical School, Dunedin New Zealand; Department of Surgery, Dunedin Hospital, Dunedin New Zealand; Department of Surgical Sciences, Otago Medical School, Dunedin New Zealand; Department of Surgery, Dunedin Hospital, Dunedin New Zealand

**Keywords:** pneumatosis coli, free air, lactulose

## Abstract

Pneumatosis coli (PC) is the radiological finding of gas within the wall of the colon. We present the case of a middle-aged woman admitted acutely with abdominal bloating and right-sided abdominal pain. Background included chronic back pain and constipation managed with long term methadone and high dose lactulose, respectively, and symptoms of abdominal bloating with increased flatulence. On abdominal examination there was right sided peritonism. Vital signs and bloods were normal. Computed tomography (CT) abdomen showed subserosal and submucosal air locules in the right colon, and a small volume of pneumoperitoneum. PC secondary to lactulose was suspected. Lactulose was ceased, and intravenous antibiotics given to suppress bacterial translocation. The patient symptomatically improved and was discharged on oral metronidazole. Her symptoms rapidly resolved and a CT scan at 6 weeks was normal. Clinically, the diagnosis of PC helped to prevent unnecessary acute surgery and allowed successful medical management to be initiated.

## Introduction

Pneumatosis coli (PC) is the radiological finding of gas within the wall of the colon. Whilst previously a rare entity, its incidence is increasing which may be partly due to the increasing use and availability of computed tomography (CT) scanning [1, 2]. PC may be idiopathic (primary)—often asymptomatic—or linked to causative pathology (secondary), such as inflammatory, infective, and ischaemic conditions, and occasionally by medications such as lactulose. The spectrum of clinical presentation is broad, ranging from an incidental finding to presentation with an acute abdomen. Management is therefore determined by patient symptoms, clinical status, and the underlying aetiology [2, 3].

## Case report

**Figure 1:**
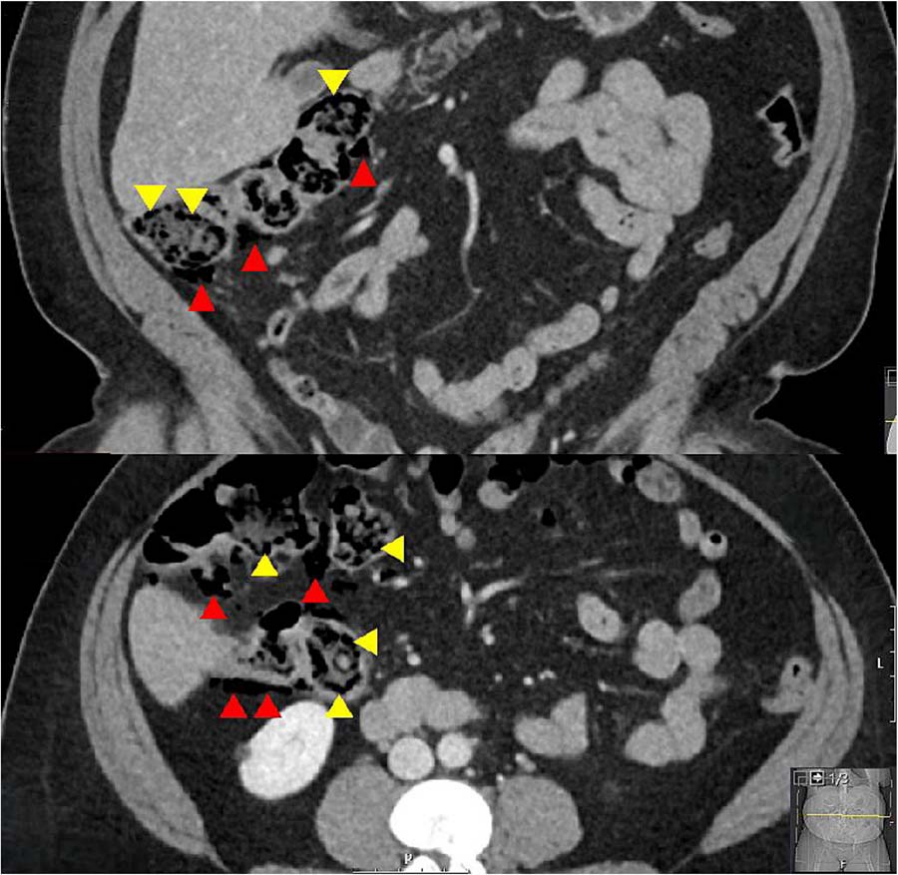
Coronal and axial CT slices showing pneumatosis in the right colon. Arrowheads demonstrate subserosal and submucosal gas.

A woman in her 50s presented to the Emergency Department with a 1-week history of worsening right sided abdominal pain. Background included chronic back pain managed with long term methadone 100 mg once daily, and previous hepatitis C treated 9 years earlier with pegylated interferon and ribavirin. There was no history of vascular disease. She was taking lactulose 50 mL once daily to treat constipation secondary to methadone and described longstanding issues with abdominal discomfort, bloating, and frequent flatulence. She was living independently with her family, was an ex-smoker and previous IV drug user. On examination she was in some distress. Her vital signs were all normal. Abdominal examination revealed peritonism in the right abdomen. Blood tests showed normal inflammatory markers with a white cell count of 6.2 × 109/L, neutrophils of 2.8 × 109/L, and a C-reactive protein (CRP) of 6 mg/L. A CT abdomen was performed and was reported to show free air, which on review showed multiple subserosal and submucosal locules of air in the caecum and ascending colon, and a small volume of intra-abdominal free air with no free fluid. There were no other features to suggest ischaemia and no colonic diverticulosis (Fig. 1).

The main differential diagnosis was between an acute abdomen with ischaemia of the right colon versus PC secondary to high dose lactulose. The degree of pain, patient distress, and localized peritonism with free air in the peritoneal cavity and air in the wall of the right colon was consistent with an acute abdomen. However, the normal vital signs and CRP of 6 mg/L suggested otherwise. The background of high dose lactulose, abdominal bloating and increased flatus, combined with the pattern of submucosal and subserosal air led to a provisional diagnosis of PC secondary to high dose lactulose being made. This resulted in a trial of medical treatment and avoidance of an acute laparoscopy or laparotomy.

**Figure 2:**
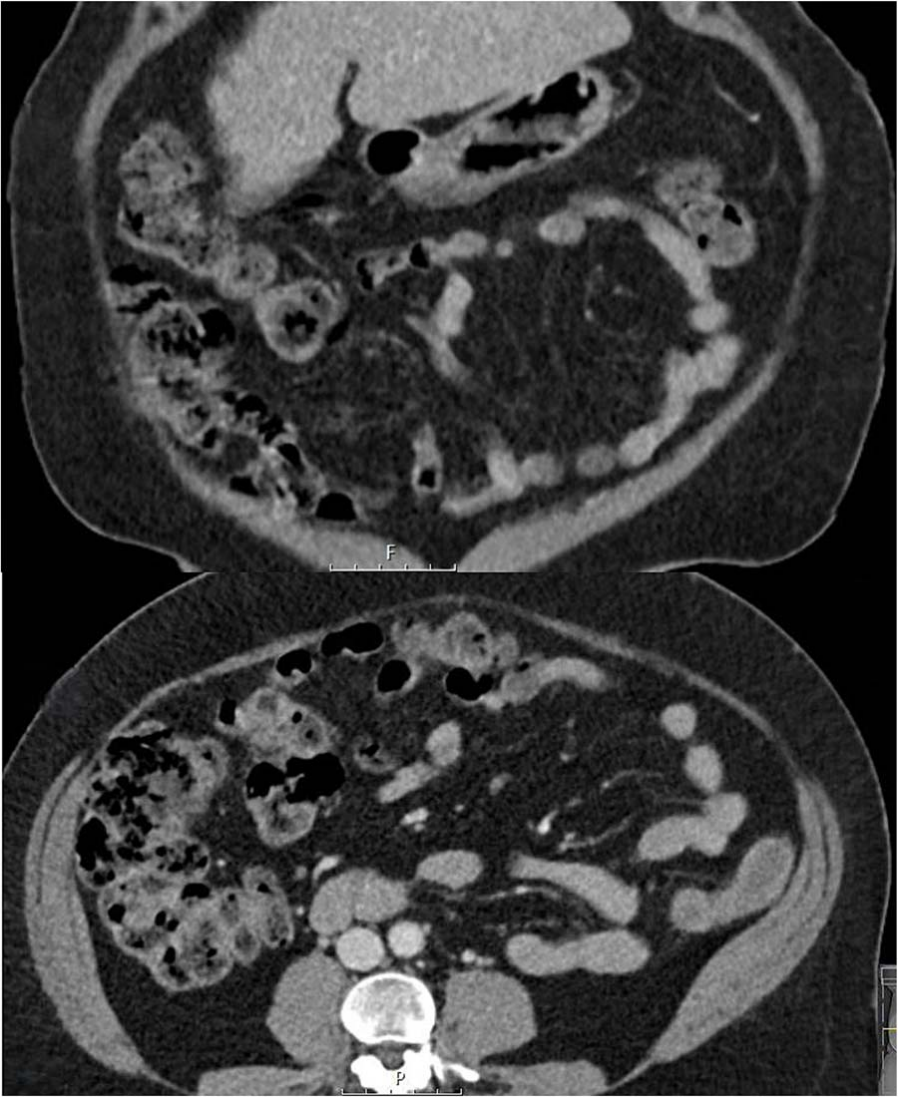
Follow up coronal and axial CT slices at 6 weeks showing resolution of PC.

Initial treatment involved serial abdominal examination to monitor progress, stopping the lactulose, and commencing IV cefuroxime 1.5 g 8-hourly and metronidazole 500 mg 12-hourly to suppress bacterial translocation. On repeat review the patient did not deteriorate. Her symptoms of abdominal pain, bloating, and flatus, and her clinical signs of peritonism were all improving within 24 h. There was ongoing clinical improvement and a repeat CT abdomen and pelvis at 48 h showed a reduction in colonic pneumatosis and pneumoperitoneum. She was discharged on a 2-week course of oral metronidazole 400 mg twice daily. Macrogol 3350 (molaxole) sachets once daily were used for treatment of her constipation instead of lactulose and the use of probiotics to maintain a healthy gut microbiome was advised. Over the subsequent 2 weeks her abdominal pain, bloating, and excessive flatulence completely resolved—for the first time in over 2 years. An interval CT scan at 6 weeks showed no evidence of pneumatosis or pneumoperitoneum (Fig. 2). At surgical outpatient clinic follow up at 3 months she remained symptom free and was grateful to no longer be troubled by her previously persistent abdominal symptoms.

## Discussion

Whilst the pathogenesis of PC is likely multifactorial three main theories have been proposed. The ``mechanical'' theory suggests that gas may enter the wall of the bowel through breaks in the mucosa or via the serosal surface by tracking along mesenteric blood vessels. The “bacterial” theory proposes that gas-forming bacteria may invade the submucosa due to increased mucosal permeability or through mucosal defects [3, 4]. The “biochemical” theory, which is consistent with our case, states that luminal bacteria can produce large volumes of hydrogen and carbon dioxide gas via fermentation of carbohydrates. As the gas pressure in the bowel lumen increases, this may diffuse into the bowel wall leading to PC [5, 6]. This may result in submucosal and subserosal cysts, with subserosal cyst rupture resulting in pneumoperitoneum, a finding reported in one in five patients with PC [7].

Lactulose, a synthetic non-absorbable disaccharide, is split by bacteria into its component monosaccharides thus providing them with a fuel source, and this effect is exacerbated by slow bowel transit. Through this breakdown process hydrogen and carbon dioxide gases are released, thus potentially leading to PC as per the “biochemical” theory [5, 6]. Methogenic and sulphate reducing bacteria, which are capable of consuming hydrogen gas, protect against PC [8]. Removing lactulose will usually result in a clinical improvement in 2–3 days [5]. Whilst PC most often involves the sigmoid and descending colon [9], in our case the right colon was predominantly affected, and this may be a feature associated with lactulose use [10].

Several cases of PC attributable to lactulose therapy have been reported previously [5, 10, 11]. These usually involve patients with established chronic liver disease and cirrhosis, and cases with pneumoperitoneum have been described. Our patient had a history of previous hepatitis C successfully treated although there was no evidence of liver cirrhosis or chronic liver disease. Clinically, the combination of high dose lactulose, increased flatulence and bloating, normal observations and bloods, and the pattern of gas on the CT supported a diagnosis of PC which could be managed medically without an acute surgical intervention. The CT finding of pericolic air, which on review was confirmed to be subsersoal air, helped to confirm the diagnosis. Cessation of lactulose and a course of oral metronidazole resulted in complete symptomatic and radiological resolution.
